# Draft Genome Sequence of Streptococcus anginosus UMB1296, Isolated from the Female Urinary Tract

**DOI:** 10.1128/MRA.00409-20

**Published:** 2020-05-14

**Authors:** Sara Temelkova, Taylor Miller-Ensminger, Adelina Voukadinova, Alan J. Wolfe, Catherine Putonti

**Affiliations:** a Neuroscience Program, Loyola University Chicago, Chicago, Illinois, USA; b Bioinformatics Program, Loyola University Chicago, Chicago, Illinois, USA; c Department of Microbiology and Immunology, Stritch School of Medicine, Loyola University Chicago, Maywood, Illinois, USA; d Department of Biology, Loyola University Chicago, Chicago, Illinois, USA; e Department of Computer Science, Loyola University Chicago, Chicago, Illinois, USA; DOE Joint Genome Institute

## Abstract

We present the draft genome sequence of a Streptococcus anginosus strain isolated from the female urinary tract. The *S. anginosus* UMB1296 draft genome has a size of 1,924,009 bp assembled into 35 contigs with a GC content of 38.69%. Genome annotation revealed 1,775 protein-coding genes, including several known virulence factors.

## ANNOUNCEMENT

Though universal consensus on its taxonomy is yet to be established, Streptococcus anginosus is commonly classified as part of the *Streptococcus milleri* group (SMG) of the genus *Streptococcus* (1, 2). Members of the SMG, which includes *S. anginosus*, S. intermedius, and S. constellatus, belong to the natural flora of human mucous membranes and healthy female urogenital tracts (2). However, they are known for their association with purulent infections throughout the body and distinct ability for causing abscesses (2, 3). Of the SMG species, *S. anginosus* has been most frequently identified from genitourinary sources (4, 5) and is largely shaped by virulence traits (6). Investigation of infections caused by *S. anginosus* have been limited, and as a result, its pathogenic potential has been historically underrecognized (7).

*S. anginosus* UMB1296 was obtained from a catheterized urine sample from a female with a urinary tract infection. The sample was clinically isolated using the expanded quantitative urinary (EQUC) protocol (8) from a prior institutional review board (IRB)-approved study (9). The genus and species for this isolate were determined via matrix-assisted laser desorption ionization–time of flight (MALDI-TOF) mass spectrometry following a previously described protocol (8). The isolate was then stored at −80°C until sequencing. The *S. anginosus* sample was streaked onto a Columbia nalidixic acid agar plate using the quadrant streaking method and a sterile inoculating loop. The plate was incubated for 24 h at 35°C with 5% CO_2_. A sterile inoculating loop was used to isolate one colony into 1 ml *Actinomyces* broth (catalog no. 40834; Millipore), and the culture was grown overnight under the same conditions as before. DNA was extracted using the Qiagen DNeasy blood and tissue kit with a modified Gram-positive extraction protocol to include the addition of 230 μl of lysis buffer (180 μl of 20 mM Tris-Cl, 2 mM sodium EDTA, and 1.2% Triton X-100 and 50 μl of lysozyme) to the culture pellet and incubation at 56°C for 10 min in a mixture of 25 μl of proteinase K and 200 μl of buffer AL. The extracted DNA was quantified using a Qubit fluorometer. DNA was sent to the Microbial Genomic Sequencing Center (MiGS) at the University of Pittsburgh for sequencing, where the DNA was first enzymatically fragmented into indices using an Illumina tagmentation enzyme. Indices were attached using PCR and sequenced using an Illumina NextSeq 550 flow cell, producing 1,765,717 pairs of paired-end reads 150 bp long. The raw reads were trimmed using Sickle v1.33 (https://github.com/najoshi/sickle) and assembled using SPAdes v3.13.0 with the “only-assembler” option for k values of 55, 77, 99, and 127 (10). Genome coverage was calculated using BBMap v38.47 (https://sourceforge.net/projects/bbmap/). While PATRIC v3.6.6 (11) was used initially to annotate the genome sequences, the NCBI Prokaryotic Genome Annotation Pipeline (PGAP) v4.11 (12) was used for reannotation and accompanies the publicly available genome. Unless previously noted, default parameters were used for each software tool.

The *S. anginosus* UMB1296 draft genome assembly is 1,924,009 bp long and is assembled into 35 contigs, with a genome coverage of 235×, a GC content of 38.69%, and an *N*_50_ score of 122,329 bp. The PGAP annotation includes 1,775 protein-coding genes. PATRIC identified 59 virulence factors, including those previously identified for the species (6), e.g., the pneumococcal surface adhesion protein PsaA. Due to the known role of the urinary microbiota in a variety of genitourinary-associated disorders, characterizing the members of this microbial community allows for further understanding of associated clinical conditions.

### Data availability.

This whole-genome shotgun project has been deposited in GenBank under the accession no. JAAUWH00000000. The version described in this paper is the first version, JAAUWH010000000. The raw sequencing reads have been deposited in SRA under the accession no. SRR11441015.

## References

[1] Jensen A, Hoshino T, Kilian M 2013 Taxonomy of the Anginosus group of the genus Streptococcus and description of Streptococcus anginosus subsp. whileyi subsp. nov. and Streptococcus constellatus subsp. viborgensis subsp. nov. Int J Syst Evol Microbiol 63:2506–2519. doi:10.1099/ijs.0.043232-0. 23223817

[2] Grinwis ME, Sibley CD, Parkins MD, Eshaghurshan CS, Rabin HR, Surette MG 2010 Characterization of Streptococcus milleri group isolates from expectorated sputum of adult patients with cystic fibrosis. J Clin Microbiol 48:395–401. doi:10.1128/JCM.01807-09. 20007382 PMC2815602

[3] Doern CD, Burnham C-A 2010 It’s not easy being green: the viridans group streptococci, with a focus on pediatric clinical manifestations. J Clin Microbiol 48:3829–3835. doi:10.1128/JCM.01563-10. 20810781 PMC3020876

[4] Whiley RA, Beighton D, Winstanley TG, Fraser HY, Hardie JM 1992 Streptococcus intermedius, Streptococcus constellatus, and Streptococcus anginosus (the Streptococcus milleri group): association with different body sites and clinical infections. J Clin Microbiol 30:243–244. doi:10.1128/JCM.30.1.243-244.1992. 1734062 PMC265033

[5] Furuichi M, Horikoshi Y 2018 Sites of infection associated with Streptococcus anginosus group among children. J Infect Chemother 24:99–102. doi:10.1016/j.jiac.2017.09.011. 29050796

[6] Sitkiewicz I 2018 How to become a killer, or is it all accidental? Virulence strategies in oral streptococci. Mol Oral Microbiol 33:1–12. doi:10.1111/omi.12192. 28727895

[7] Asam D, Spellerberg B 2014 Molecular pathogenicity of Streptococcus anginosus. Mol Oral Microbiol 29:145–155. doi:10.1111/omi.12056. 24848553

[8] Hilt EE, McKinley K, Pearce MM, Rosenfeld AB, Zilliox MJ, Mueller ER, Brubaker L, Gai X, Wolfe AJ, Schreckenberger PC 2014 Urine is not sterile: use of enhanced urine culture techniques to detect resident bacterial flora in the adult female bladder. J Clin Microbiol 52:871–876. doi:10.1128/JCM.02876-13. 24371246 PMC3957746

[9] Price TK, Dune T, Hilt EE, Thomas-White KJ, Kliethermes S, Brincat C, Brubaker L, Wolfe AJ, Mueller ER, Schreckenberger PC 2016 The clinical urine culture: enhanced techniques improve detection of clinically relevant microorganisms. J Clin Microbiol 54:1216–1222. doi:10.1128/JCM.00044-16. 26962083 PMC4844725

[10] Bankevich A, Nurk S, Antipov D, Gurevich AA, Dvorkin M, Kulikov AS, Lesin VM, Nikolenko SI, Pham S, Prjibelski AD, Pyshkin AV, Sirotkin AV, Vyahhi N, Tesler G, Alekseyev MA, Pevzner PA 2012 SPAdes: a new genome assembly algorithm and its applications to single-cell sequencing. J Comput Biol 19:455–477. doi:10.1089/cmb.2012.0021. 22506599 PMC3342519

[11] Brettin T, Davis JJ, Disz T, Edwards RA, Gerdes S, Olsen GJ, Olson R, Overbeek R, Parrello B, Pusch GD, Shukla M, Thomason JA III, Stevens R, Vonstein V, Wattam AR, Xia F 2015 RASTtk: a modular and extensible implementation of the RAST algorithm for building custom annotation pipelines and annotating batches of genomes. Sci Rep 5:8365. doi:10.1038/srep08365. 25666585 PMC4322359

[12] Tatusova T, DiCuccio M, Badretdin A, Chetvernin V, Nawrocki EP, Zaslavsky L, Lomsadze A, Pruitt KD, Borodovsky M, Ostell J 2016 NCBI Prokaryotic Genome Annotation Pipeline. Nucleic Acids Res 44:6614–6624. doi:10.1093/nar/gkw569. 27342282 PMC5001611

